# Robust Removal of Slow Artifactual Dynamics Induced by Deep Brain Stimulation in Local Field Potential Recordings Using SVD-Based Adaptive Filtering

**DOI:** 10.3390/bioengineering10060719

**Published:** 2023-06-14

**Authors:** Nooshin Bahador, Josh Saha, Mohammad R. Rezaei, Saha Utpal, Ayda Ghahremani, Robert Chen, Milad Lankarany

**Affiliations:** 1 Krembil Research Institute, University Health Network (UHN), 60 Leonard Ave, Toronto, ON M5T 0S8, Canada; nooshin.bahador@uhnresearch.ca (N.B.);; 2 Institute of Biomedical Engineering (BME), University of Toronto, Toronto, ON M5S 2E8, Canada; 3 Department of Electrical and Computer Engineering, University of Waterloo, Toronto, ON N2L 3G1, Canada; 4 School of Medicine, Stanford University, Stanford, CA 94305, USA; 5 Department of Medicine, Division of Neurology, University of Toronto, Toronto, ON M5S 2E8, Canada; 6 KITE Research Institute, Toronto Rehabilitation Institute, University Health Network (UHN), Toronto, ON M5G 2A2, Canada; 7 Department of Physiology, University of Toronto, Toronto, ON M5S 2E8, Canada

**Keywords:** deep brain stimulation (DBS), DBS artifact, adaptive filtering, Stroop task, local field potentials (LFP)

## Abstract

Deep brain stimulation (DBS) is widely used as a treatment option for patients with movement disorders. In addition to its clinical impact, DBS has been utilized in the field of cognitive neuroscience, wherein the answers to several fundamental questions underpinning the mechanisms of neuromodulation in decision making rely on the ways in which a burst of DBS pulses, usually delivered at a clinical frequency, i.e., 130 Hz, perturb participants’ choices. It was observed that neural activities recorded during DBS were contaminated with large artifacts, which lasts for a few milliseconds, as well as a low-frequency (slow) signal (~1–2 Hz) that can persist for hundreds of milliseconds. While the focus of most of methods for removing DBS artifacts was on the former, the artifact removal capabilities of the slow signal have not been addressed. In this work, we propose a new method based on combining singular value decomposition (SVD) and normalized adaptive filtering to remove both large (fast) and slow artifacts in local field potentials, recorded during a cognitive task in which bursts of DBS were utilized. Using synthetic data, we show that our proposed algorithm outperforms four commonly used techniques in the literature, namely, (1) normalized least mean square adaptive filtering, (2) optimal FIR Wiener filtering, (3) Gaussian model matching, and (4) moving average. The algorithm’s capabilities are further demonstrated by its ability to effectively remove DBS artifacts in local field potentials recorded from the subthalamic nucleus during a verbal Stroop task, highlighting its utility in real-world applications.

## 1. Introduction

The impact of deep brain stimulation (DBS) on local field potential (LFP) recordings can vary depending on the pattern of pulses used [1,2,3]. Recent research revealed that multi-pulse stimulation-induced artifacts can persist for up to 1.5 milliseconds [1]. However, earlier studies have found that DBS artifacts can contaminate recorded signals for much longer periods, potentially lasting tens, or hundreds of milliseconds [2]. Additionally, another work noted that the shape of DBS artifacts can be influenced by factors such as the shape and impedance of electrodes, the distance between the stimulation and recording sites, as well as the amplitude, frequency, and duration of stimulus pulses [3].

Several filtering techniques have been proposed for removing artifacts from LFP recordings in the literature. These include a low-pass filter [4], a notch filter [5], and band-pass filters [6]. Specifically, reference [4] focused on removing high-frequency artifacts generated by DBS (130 Hz) by implementing a low-pass filter with a corner frequency of 50 Hz using Butterworth coefficients. Another study [5] reported that no biomarkers for Parkinson’s disease were found in the frequency band between 125 and 155 Hz and thus a high-order (8th) Chebyshev notch filter with a center frequency of 140 Hz and a stopband between 125 and 155 Hz was used to suppress artifacts during DBS. A band-pass filter with a frequency band between 100 Hz and a Nyquist frequency of 211 Hz was used to eliminate all stimulation interference [6].

Template subtraction is a commonly used method for eliminating DBS artifacts from LFP recordings. Several studies have explored different methods of implementing this technique [7]. They used thresholding to identify DBS events and then located segments of the LFP that may have been affected by artifacts by analyzing the minimum and zero-crossing points surrounding each detected event. They then employed an ensemble empirical mode decomposition (EEMD) algorithm to decompose the segment into intrinsic mode functions (IMFs) and reconstructed the DBS template through a weighted summation of these IMFs, with greater weight given to IMFs with higher frequencies. Another work [8] took a different approach, first detrending the LFP signal by eliminating the residue of the IMF set, then segmenting DBS pulses based on peak points, creating an average artifact shape derived from all pulses, which was then smoothed using a moving average filter to reconstruct the artifact template. Similarly, the averaging all the stimulus artifact segments, and a short time period of 0.4 ms around them, was used to construct the template of the stimulus artifact [9]. Although template matching is an effective method to identify specific patterns of interest, the variability of DBS artifacts may introduce additional noise into the data, making it difficult to accurately match templates and identify the desired signal.

A technique for removing DBS artifacts from LFP recordings, known as detrending, uses empirical mode decomposition (EMD) [10]. The approach involved identifying the last intrinsic mode function (IMF) as a trend and removing it to generate a detrended signal. DBS events were then considered as a waveform composed of a set of sine waves, and the amplitude, frequency, and phase of these waves were initialized by visually inspecting the power spectrum. These parameters were then iteratively removed from each segment of the contaminated recording. Another method of removing DBS artifacts is the dynamic averaging of small groups of consecutive segments [11,12]. Segments that are temporally close to each other have similar DBS artifact shapes, and therefore, artifacts can be removed by taking the average of small groups of consecutive segments [11,12]. Similarly, DBS pulses were detected by amplitude thresholding, and took an average of neighboring segments before and after each pulse to generate the stimulus artifact template, which was then subtracted from the raw data at the location of each pulse [13,14].

Another method for filtering DBS artifacts from LFP recordings is based on temporal decomposition, using mutual information between independent components and the reference signal of the DBS [15,16]. The signals were decomposed into temporally independent components using independent component analysis (ICA) and then the mutual information (MI) was computed, between the DBS artifact and each of these individual components, to determine how much information is shared between them. Finally, components that showed high mutual information were removed. Another technique for removing DBS artifacts is space separation to extract artifact subspace [17,18,19,20]. This approach assumes that the DBS component and the original signal are approximately orthogonal to each other, thus, the raw signal is projected onto the orthogonal components using signal space projection (SSP), and the DBS artifact is identified and projected out. Eigenvalue decomposition (EVD) of the covariance matrix computed from concatenated epochs, including DBS events, was performed and then projected onto orthogonal signal and artifact subspaces. The segments, including DBS artifacts, were then projected out of the noise subspace. Another study [21] also extracted eigenvectors that span the subspace of the DBS artifact at a specific frequency.

Principle component analysis (PCA) was also employed to identify orthogonal bases that effectively captured the majority of the variance in the data, and to eliminate eigenspaces associated with artifacts [22]. They demonstrated that this approach was more effective at removing deep brain stimulation (DBS) artifacts than alternative techniques such as signal space separation. A different method was employed using thresholding to detect DBS artifact peaks and, subsequently, setting samples within the affected period to zero [23]. The missing data were then interpolated by utilizing the neighboring samples that preceded and followed the contaminated period. Other solutions proposed to address the issue of DBS artifacts in LFP recordings include: adaptive filtering [24]; linear Wiener filtering [25]; template subtraction using an adaptive shape based on the Euclidean median of k-nearest neighbors [26]; weighted moving average template subtraction, in which the template is estimated as a weighted average of a limited number of neighboring pulses [27]; a template-based subtraction method that utilizes past artifact samples and linear regression [28]; interpolation techniques, including linear proposed [29], Gaussian [30], and Cubic Spline [31]; a symbiotic combination of front-end and back-end template subtraction [32]; and polynomial subtraction of power spectral density in the frequency domain to mitigate low-frequency distortions in LFP arising from impedance mismatch during DBS therapy [33]. Despite the effectiveness of these techniques in filtering unwanted noise, there is always a risk of over-filtering, which means that these methods may remove not only unwanted noise but also important information from the signal. This can be particularly problematic for slow wave activities in LFP signals, which can contain important information about brain activity and function.

A summary of limitations of the state-of-the-art methods is provided in Table 1.

As previously mentioned, methods such as template matching may introduce additional noise to the data due to the varying nature of DBS artifacts. While techniques such as adaptive filtering, FIR Wiener filtering, and moving average techniques can effectively preserve fast oscillations, there is a risk of over-filtering, which may result in the removal of important information from the critical slow wave activities in LFP signals. In the present study, we develop a hybrid filtering method that utilizes the strengths of multiple techniques, including SVD and adaptive filtering, to eliminate both sharp spikes and slow wave artifacts.

## 2. Materials and Methods

**Figure 1:**
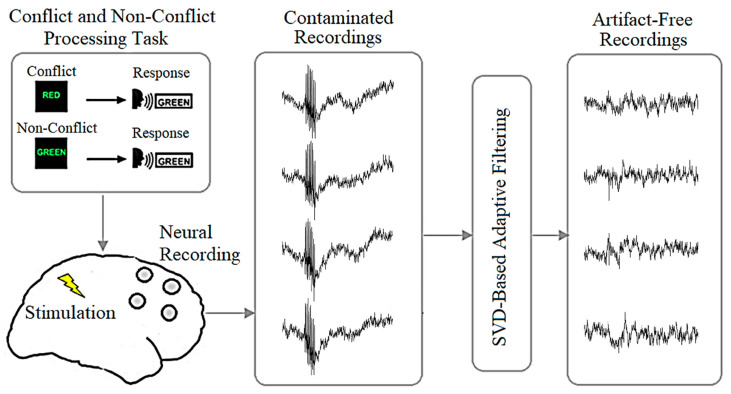
A Conceptual Framework for SVD-based Adaptive Filtering of DBS-Induced Artifacts during a Verbal Stroop Task: This figure illustrates the conceptual framework of the SVD-based adaptive filtering algorithm for removing both high- and low-frequency artifacts induced by deep brain stimulation (DBS) during a verbal Stroop task. The final window in the figure presents the outcome of the algorithm, where we can see the signal without DBS artifact, and therefore yielding a more accurate measurement of neural activity related to cognitive processes.

The proposed SVD-based adaptive filtering technique for removing DBS-induced slow and fast dynamics is conceptually illustrated in Figure 1. These dynamics can greatly impact the processing of LFP and EEG signals during cognitive tasks. In this study, data recorded from patients with Parkinson’s disease (PD) performing a verbal Stroop task were used. As demonstrated in Figure 1, DBS modulates the neural activities of subthalamic neurons, while LFP and EEG signals are recorded from multiple electrodes. Through the application of the SVD-based adaptive filtering algorithm, the artifacts observed during and after DBS pulses were effectively removed. (See Section 2.2.1 for further information on the experimental data).

### 2.1. Data

The present study includes an examination of both synthetic and experimental data sets.

#### 2.1.1. Synthetic Data

**Figure 2:**
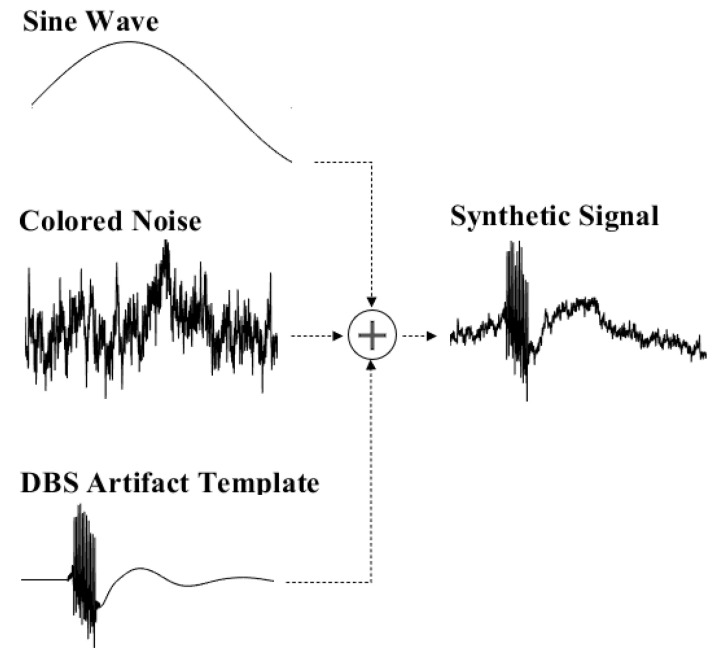
Synthetic Signal Generation Process: This figure illustrates the procedure for creating synthetic signals, which consists of a combination of a sine wave representing slow-wave movement artifacts, colored noise representing neural activity, and DBS artifacts modeled on a stimulus artifact template.

The synthetic dataset comprised a sine wave, colored noise, and a DBS artifact template (as illustrated in Figure 2). The synthetic signal had a duration of 3 s and was sampled at a frequency of 1.5 kHz. To create the contaminated signal, both colored noise and a sine wave were added to the artifact template. The colored noise had a weight of 10, while the sine wave had a weight of 40.

To generate colored noises, a white noise sequence is passed through an auto-regressive (AR) filter with an order of N [34]:

(1) $$\sum_{k=0}^{k=N}a_{k}y\left[n-k\right]=x[n]$$

where x[n] is a white noise.

The AR filter coefficients can be produced as [34]:

(2) $$\begin{array}{c} a_{0}=1\\ a_{k}=\left(k-1-\frac{\alpha }{2}\right)\frac{a_{k}-1}{k} \end{array}$$

which can be employed as an infinite impulse response (IIR) filter by utilizing the filter transfer function in the following format [34]:

(3) $$H\left(z\right)=\frac{1}{1+\sum_{k=1}^{N}a_{k}z^{-k}}$$

The “dsp.ColoredNoise” class was used in Matlab software to execute this process.

The concept of combining a sine wave and colored noise originated from a PhD thesis conducted at the Institute of Biomaterials and Biomedical Engineering, University of Toronto. The thesis explored the generation of synthetic EEG signals by introducing Gaussian noise to a sine wave [35]. Additionally, the sine wave can serve as a simulation for a low-frequency LFP [36].

#### 2.1.2. Experimental Data

For the experimental portion of the study, data were collected from patients with Parkinson’s disease who were receiving trains of DBS during specific periods of a Stroop task (nine men, with an average age of 60.4 years and an age range of 44 to 73 years). The task involved displaying a color word on a screen and asking the patients to name the ink color of the word into a microphone. In the conflict trials, the word and ink color did not match, while in the non-conflict trials, they did match [37].

Four different stimulation conditions were used, with each condition applied randomly to one trial out of every four. These four conditions were: No stimulation, Ready period, Early response, and Late response. Each patient completed a total of 240 trials, with event-related stimulation applied during each trial after a certain number of test trials had been completed. Bilateral subthalamic nucleus (STN) LFPs and scalp EEG were recorded during the trials, using a sampling frequency of 5 kHz and a filtering range of 1–1000 Hz. The data were down-sampled to 1000 Hz for analysis. Only trials with response times between 0.3 and 1.8 s were included in the analyses [37].

The trains of pulses used for stimulation consisted of 11 pulses with a duration of approximately 80 milliseconds, a frequency of 130 Hz, a monophasic waveform, and a pulse width of 100 microseconds. The stimulation intensity for event-related deep brain stimulation (DBS) was 1.92 ± 0.76 milliamperes (mA). The trigger pulses for stimulation were generated using Spike2 software from Cambridge Electronic Design in the UK, and were delivered through a constant current stimulator from Digitimer in Welwyn Garden City, Hertfordshire, UK [37].

Neurophysiological mapping was employed to confirm the stimulated contact’s location. Intraoperative recordings were used to identify the ventral border of the STN by detecting a change in the pattern of cell firing, which was then used to determine the final target for the DBS electrode implantation. The STN’s top and bottom margins were determined with reference to the DBS electrode’s final position (DBS 3387 electrode, Medtronic, Minneapolis), as reported in the operative notes. The location of each contact relative to the STN was estimated using the electrode’s known dimensions, i.e., four 1.5mm contacts spaced 1.5 mm apart [37].

For better understanding of the experiment’s details and other relevant information about the dataset, we strongly recommend referring to the published article by the research team led by Dr. Robert Chen at Toronto Western Hospital [37].

### 2.2. Benchmark DBS-Artifact Suppression Techniques in this Study

For the comparison of the performance of the proposed technique, four existing methods were used as benchmark techniques. These methods were normalized least mean square adaptive filtering [38], optimal FIR Wiener filtering [39], Gaussian model matching [40], and moving average techniques [27].

#### 2.2.1. Normalized Least Mean Square (NLMS) Adaptive Filter Algorithm

The main structure of a NLMS filter consists of a FIR filter with a coefficient update procedure in which the coefficients are updated so that the difference between the output and the reference signal becomes minimum.

The NLMS filter is defined by the following equations.

(4) $$\begin{array}{c} y\left[n\right]=w^{T}\left[n-1\right]u[n]\\ e\left[n\right]=d\left[n\right]-y[n]\\ w\left[n\right]=\alpha w\left[n-1\right]+f\left(u\left[n\right],e\left[n\right],\mu \right)\\ f\left(u\left[n\right],e\left[n\right],\mu \right)=\mu e\left[n\right]\frac{u^{*}[n]}{{\varepsilon +u}^{H}\left[n\right]u[n]} \end{array}$$

where *n* is the current index of temporal sample, *u* is the input signal, $u^{*}$ is the complex conjugate of input signal, $u^{H}$ is the conjugate transpose of input signal, *w* is the filter weight, y is the output signal, *e* is the estimation error, *d* is the reference signal, μ is the adaptation step size, and ε is the stability constant [41].

#### 2.2.2. Optimal FIR Wiener Filter

FIR Wiener filter produces an estimation of the artifact using a reference signal. The optimal filter satisfies the Wiener–Hopf equation given by following equation [42]:

(5) $$RH=R_{xy}$$

where *R*, *H* and $R_{xy}$ are, respectively, the symmetric Toeplitz autocorrelation matrix of the input signal, impulse response of the optimal filter, and cross-correlation between the input and the desired output. The matrix of *R* is of the form given by:

(6) $$R=\left(\begin{array}{ccc} r_{1} & \cdots & r_{n}\\ \vdots & \ddots & \vdots \\ r_{n} & \ldots & r_{1} \end{array}\right)$$

#### 2.2.3. Gaussian Model Matching

The global peaks in the signal are fitted using Gaussian model which is given by:

(7) $$y=\sum_{i=1}^{k}\alpha_{i}e^{\left[-{\left(\frac{x-\beta_{i}}{\gamma_{i}}\right)}^{2}\right]}$$

where α, β, γ and *k* are, respectively, the amplitude, centroid, peak width, and the number of peaks to fit.

This fitted model is then used as the artifact template and subtracted from the original signal to provide a clean version of the signal.

#### 2.2.4. Moving Average

The artifact template is generated based on the local *k*-sample average values, where each average is calculated over a sliding window of length k across neighboring samples of original signal. The window of length k is shifted across the neighboring samples of the original signal. At each position of the window, the k samples within the window are considered and the average is calculated. By applying this iteratively for different positions of the sliding window across the original signal, the artifact template based on the local k-sample average values is generated.

(8) $$\mu =\frac{1}{k}\sum_{i=1}^{k}f_{i}$$

The filtered signal is then obtained by template subtraction from the original signal.

### 2.3. Proposed Algorithm

The SVD adaptive filtering algorithm is a method for removing slow components of DBS artifacts from an input signal while also reducing the presence of sharp DBS peaks. This filtering includes two separate modules for removing slow and fast waves of DBS artifacts (DBS artifacts start with fast and sharp transients followed by a slow wave).

**Figure 3:**
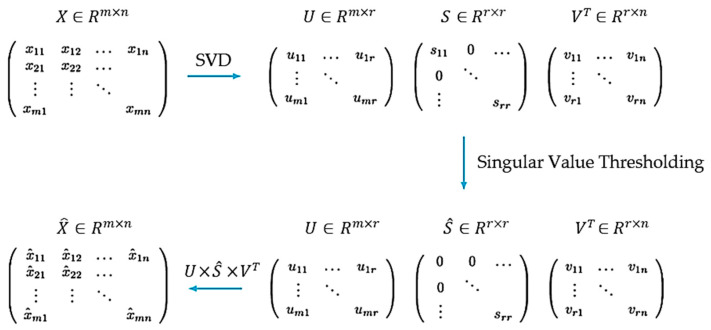
Signal reconstruction based on singular value decomposition.

**Figure 4:**
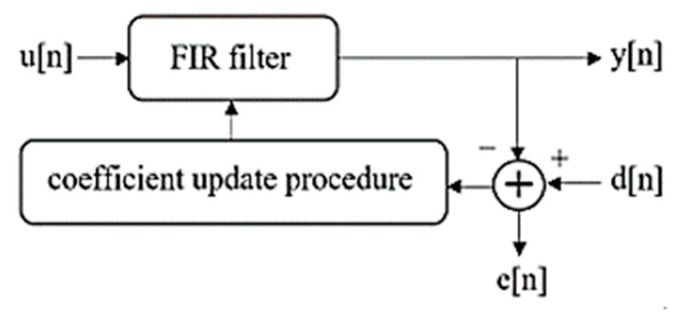
Adaptive filtering procedure.

The process begins by initializing the input signal. The algorithm then generates a set of circularly shifted versions of the input signal by repeatedly shifting the elements a certain number of positions. These shifted signals are stored and then combined to create a matrix. Next, the algorithm performs SVD to obtain the matrices *U*, *S*, and *V*. The diagonal matrix *S* is extracted, and any singular values that are more than 90% of the maximum singular value are set to zero (Figure 3). Using the modified *S* matrix, along with *U* and *V*, the algorithm reconstructs the signal. A reference signal is then calculated by finding the moving average of the corresponding values, with a window size of 20. Starting from the DBS time until the end of the array, the values of reconstructed signal are replaced with the reference signal. An adaptive filtering algorithm using the least mean squares (LMS) method is then applied to extract the high frequency component and suppress the DBS peaks (Figure 4). This process is repeated twice more to further reduce the amplitude of these peaks.

Here is the Algorithm 1 that describes the above steps in the proposed technique:
**Algorithm 1:** SVD Adaptive FilteringInitialize a variable *X* as the input signal.Generate a set of circularly shifted versions of the input signal by looping through a range of 1 to 40, and for each iteration:
Circularly shift the elements of *X* by the current iteration number of positions.Append the shifted signal to a new variable *X^shifted^*.Concatenate the original signal *X* and the shifted versions in *X^shifted^* to form the signal matrix *X^concat^*.Perform SVD on the concatenated signal matrix *X^concat^* to obtain the matrices *U*, *S*, *V*.Extract the diagonal matrix S from the SVD results.Zero out the largest singular values in the S matrix, which are determined by values greater than 90% of the maximum singular value.Use the modified *S* matrix, along with the *U* and *V* matrices from the SVD, to reconstruct the signal matrix *X^recon^*.Compute a reference signal using the moving average of the corresponding values with a window size of 20.Starting from DBS time to the end of the array, replace the values of *X^recon^* with the reference signal.Implement an adaptive filtering algorithm using the least mean squares (LMS) method to extract a high frequency component and suppress the sharp DBS peaks:Apply a moving average filter to the input signal *X* with a window size of 100.Initialize an LMS filter object with filter length of 7 and step size of a small value.Apply the LMS filter to the *X^recon^* signal to obtain the filter weights and error signal.Locate two change points in the error signal, assuming them to correspond to the period including sharp DBS peaks.Locate the peaks in the error signal within these two change points and reduce their amplitudes by a factor of 10.Repeat the process twice more to further reduce the amplitudes of these peaks.

## 3. Results

### 3.1. Validation of Artifact Removal Algorithms on Synthetic LFP Signal

**Figure 5:**
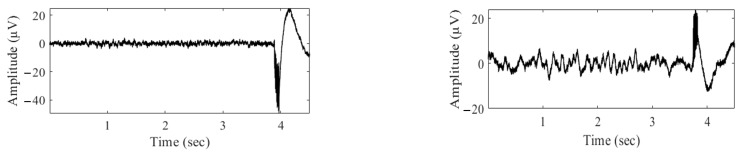
The effect of reversing the polarity of the stimulus on the slow wave component of DBS artifacts; when the stimulus is reversed, the artifact is reversed or mitigated.

Figure 5 presents a comparison of the contaminated signals for positive and negative pulses. The first sample (left) illustrates the contaminated signal when the pulse is negative, while the second sample (right) shows the contaminated signal when the pulse is positive. This comparison highlights the effect of DBS pulse polarity on the contamination of the signal.

**Figure 6:**
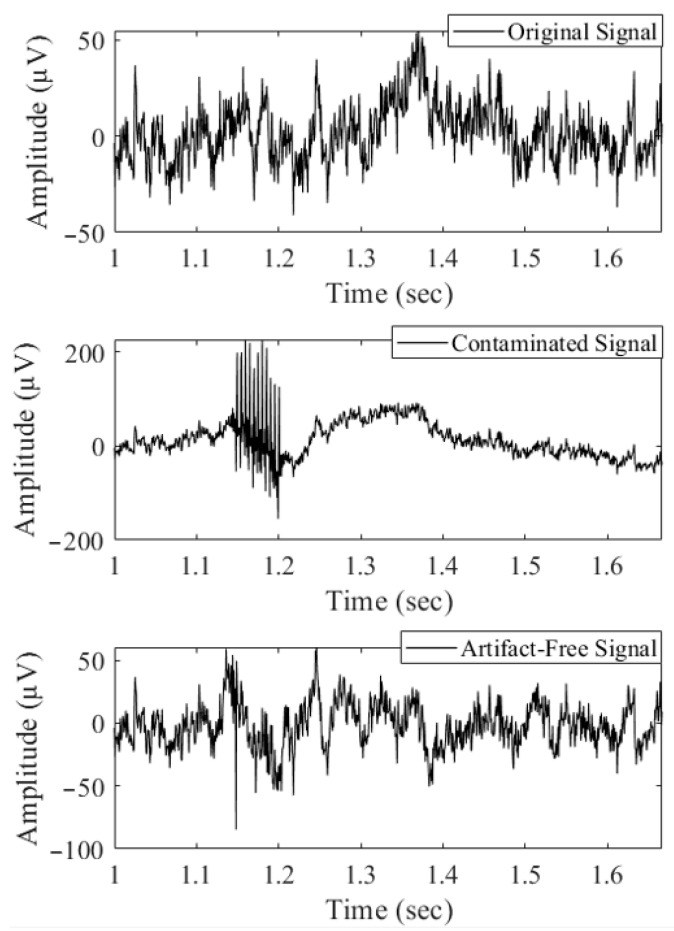
Comparing the clean synthetic signal after artifact removal to the original and contaminated synthetic signals.

The time–frequency analysis in Figure 6 was conducted to assess the preservation of the original signal’s temporal and spectral information after DBS artifact removal. The availability of the clean version of synthetic signals made it easy to evaluate the performance of the artifact removal algorithm. By visually comparing the time-domain signals before and after the removal of DBS artifacts, it is evident that the algorithm not only effectively eliminated the sharp spikes caused by DBS, but also the slow activities induced by both the DBS artifact and sine wave. Additionally, by observing the small fluctuations in the amplitude of the original signal, it can be seen that the filtered version closely resembles the original signal. The comparison of the spectrogram plots, in Figure 6, between the original and artifact-free signals confirms the preservation of temporal–spectral patterns within the original signal and demonstrates the good performance of the proposed filtering technique.

Table 2 quantitatively evaluates the quality of filtering by comparing the differences in average power between original and contaminated signals with artifact-free signals in various frequency bands. The percentages of reduction in differences are as follows: Delta: 54.24%, Theta: 69.10%, Alpha: 84.32%, and Beta: −43.75%.

The mean-squared error (MSE) between the original synthetic and contaminated signals was compared to the MSE between the original synthetic and artifact-free signals in time domain (Table 3). The percentage difference obtained by SVD filtering was 95.26%, which outperformed other techniques. Adaptive filtering came in second place with a percentage difference of 46.99%. Based on these results, it was hypothesized that combining SVD with adaptive filtering may improve the suppressing of both DBS and motion artifacts. This hypothesis was tested on real data and the results are presented in following subsection.

### 3.2. Validation of Artifact Removal Algorithms on Experimental LFP Signal

**Figure 7:**
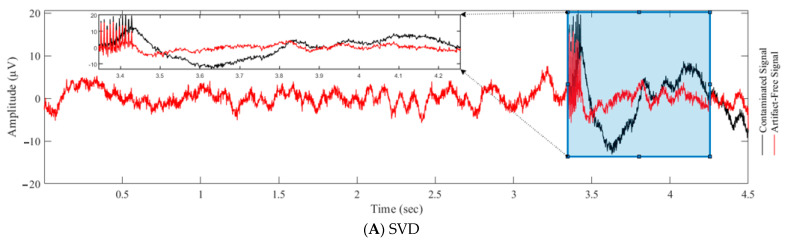

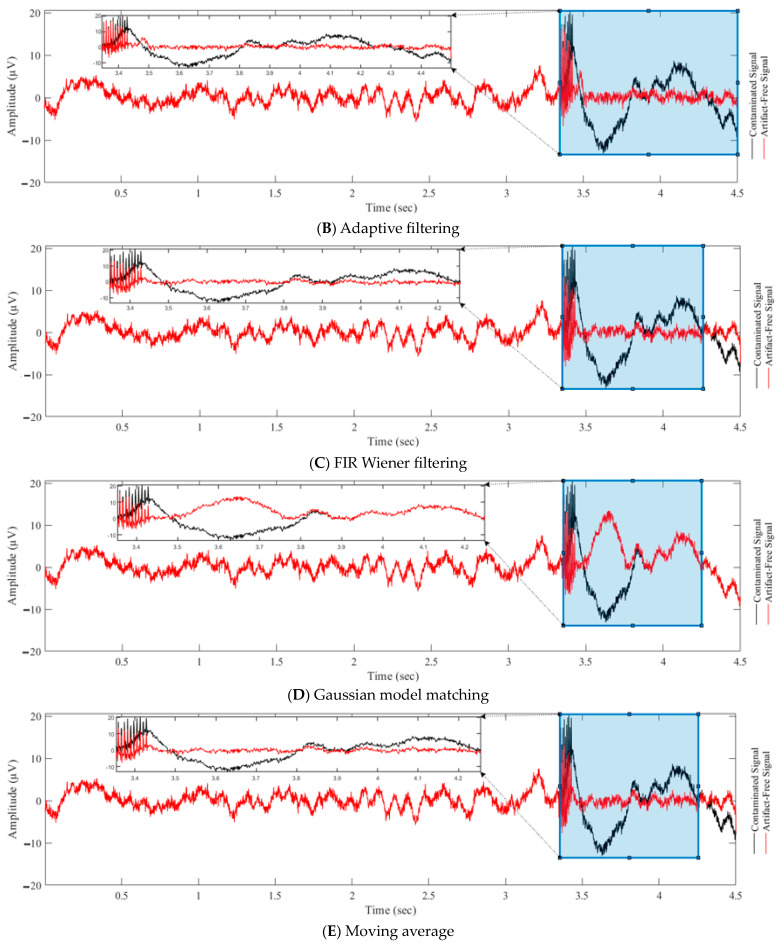
Evaluation of various techniques for suppressing artifacts in DBS signals. The aim of this comparison was to identify the most effective combination of techniques for eliminating both sharp spikes and slow waves caused by DBS pulses. A comprehensive analysis of different filtering methods was considered to provide insight into ways to improve the overall quality of DBS filtering.

Figure 7 displays the evaluation of various filtering techniques, including SVD (Figure 7A), adaptive filtering (Figure 7B), FIR Wiener filtering (Figure 7C), Gaussian model matching (Figure 7D), and moving average (Figure 7E), for suppressing artifacts in DBS signals, with the aim of identifying the most effective combination of techniques for eliminating sharp spikes and slow waves caused by DBS pulses. Figure 7E shows that the use of a moving average window smoothed out most slow activities, including non-artifactual components, and yet sharp spikes induced by DBS remained present in the filtered signal. Similarly, adaptive filtering in Figure 7B and FIR wiener filtering in Figure 7C flattened out artifactual and non-artifactual slow activities without removing sharp spikes. In contrast, the use of a Gaussian model matching filter in Figure 7D introduced unwanted noise into the signal. It is clear from these results that the use of a single technique, such as moving average filtering, adaptive filtering, or FIR wiener filtering, is not sufficient to effectively remove both fast and slow artifactual components without affecting the useful neural information. Instead, the most effective approach would be to utilize the strengths of multiple techniques, such as the SVD technique (Figure 7A) for reproducing clean slow wave activities in the original signal and adaptive filtering for reproducing fast oscillations. In this way, both sharp spikes and slow wave artifacts induced by DBS can be effectively eliminated.

Table 4 demonstrates that the average power of the filtered signal using SVD is closer to the baseline value within the delta and theta frequency bands, when compared to other techniques. This highlights the capability of SVD in reconstructing the slow wave activities present in the original signal.

**Figure 8:**
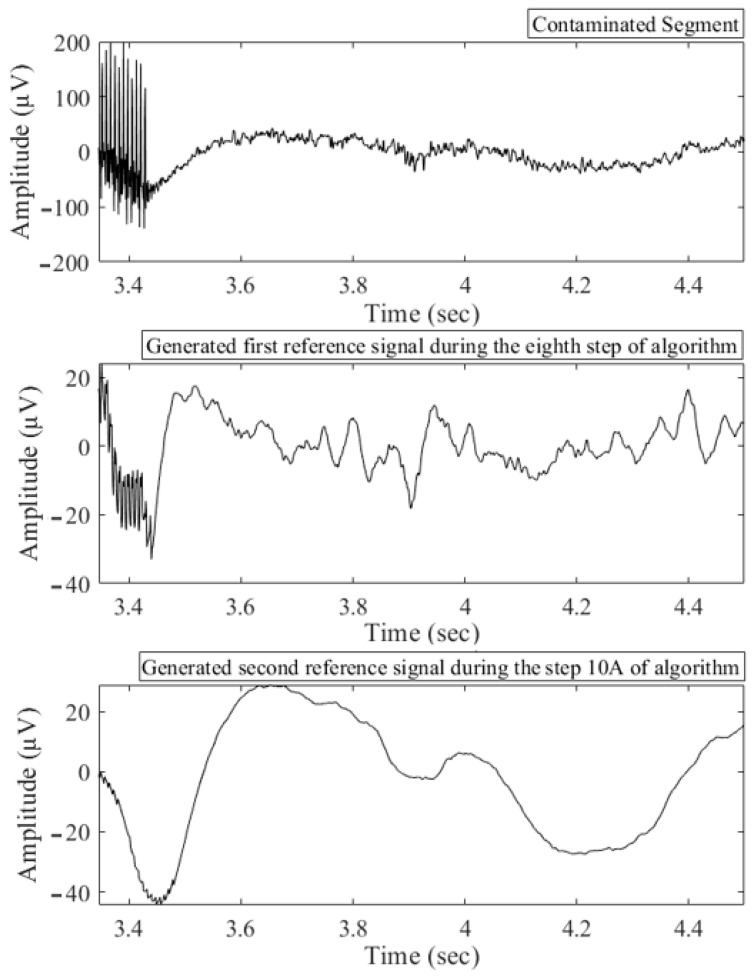
Contaminated Signal and Reference Signals Generated in Different Steps.

**Figure 9:**
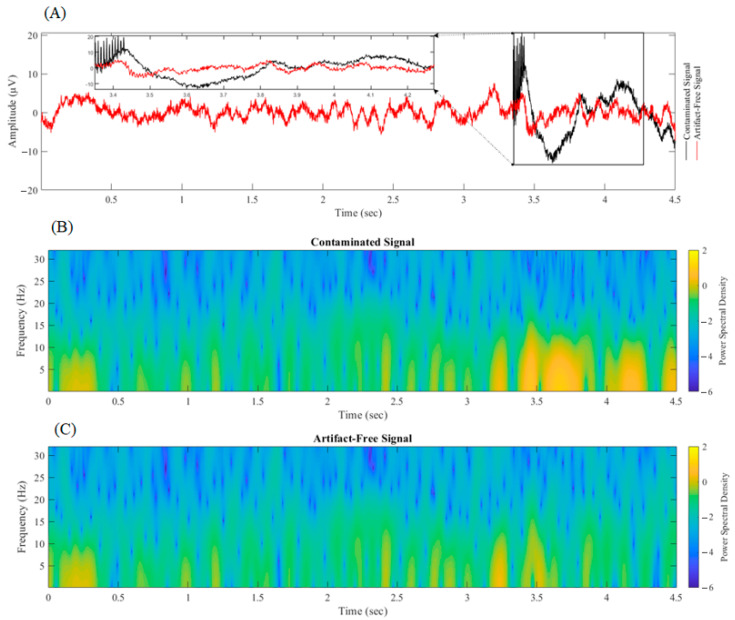
SVD-based Adaptive Filtering for DBS Artifact Removal: This figure illustrates the effectiveness of combining SVD with adaptive filtering for removing deep brain stimulation (DBS) artifacts. The subplot (**A**) compares the contaminated signal with the filtered version in the time domain, with the enlarged window highlighting the beginning of DBS. The subplots (**B**,**C**) provide a spectral analysis of the contaminated and filtered signals, respectively, through the use of spectrograms. The SVD-based adaptive filtering technique is able to effectively remove DBS artifacts while preserving the underlying neural activity, as demonstrated by the clean version of the signal and the consistent spectral content in the filtered version of the signal.

Figure 8 illustrates the reference signals generated at various stages of the proposed algorithm. Figure 9 presents the results of an SVD-based adaptive filtering technique applied to remove DBS artifacts. The subplot A of the figure demonstrates the effectiveness of the technique in preserving neural activity while effectively filtering out the sharp spikes and slow bumps associated with DBS. The low and high frequency components induced by neural activity remain untouched, while the DBS artifacts are successfully removed. The subplots of B and C provide additional support for the technique’s effectiveness through the use of spectrograms. A spectrogram is a visual representation of the frequency content of a signal over time, and it provides a powerful tool for analyzing and interpreting signals, particularly those that vary over time. The spectrograms in these subplots demonstrate the effectiveness of the SVD-based adaptive filtering technique in removing the slow wave activities induced by DBS. By eliminating these artifacts, the spectrograms reveal a clean and accurate representation of neural activity. Specifically, the spectral content of the filtered signal is seen to closely match the spectral content of the original neural activity, with a significant reduction in the spectral content of the DBS artifacts. This is a strong indication that the SVD-based adaptive filtering technique is effectively removing these artifacts, while preserving the underlying neural activity.

## 4. Conclusions

Neurostimulation has emerged as a cutting-edge technology for the treatment of neurological disorders, offering great potential for closed-loop neuromodulation. However, recorded neural signals, such as LFPs, are often contaminated by artifacts induced by stimulation pulses. We observed that a short DBS burst induced both fast (sharp, lasting for a few msec) and slow (lasting for hundreds of msec) artifacts.

Here, we proposed a solution using SVD-based adaptive filtering. To address the issue of over filtering with adaptive filtering, an additional module was included in the study to separate meaningful slow wave activities from artifacts. This module reproduced the slow wave activities in LFP recordings by applying the SVD and considering singular values less than a specific threshold. Our synthetic results showed that the proposed technique exhibits a superior performance compared to existing methods, and successfully recovered both high and low-frequency neural information without increasing noise levels. Using LFPs recorded from STN during a verbal Stroop task, we demonstrated that SVD-adaptive filtering successfully removed slow artifactual dynamics induced by a short burst of DBS.

The proposed adaptive filtering approach improved the detection of neural oscillations in a short time period after DBS pulses which, in turn, can be useful for enhancing the efficacy of closed-loop neuromodulation devices. By effectively suppressing DBS artifacts from LFP recordings, we anticipate that the proposed SVD-based adaptive filtering method will provide a valuable tool for researchers and practitioners in the field of neurostimulation.

One potential limitation of our proposed technique is the lack of real-time adaptation. It may have a high computational cost, especially when applied to large-scale datasets. Although adaptive filtering is known for its ability to adapt to changes in signal power and statistical properties, the combined approach may not be able to handle rapid changes in real-time scenarios. In future work, we will improve the implementation of the algorithm for real-time applications.

Our objective for future works is to enhance the efficiency of our methodology in the real-time removal of deep brain stimulation (DBS) artifacts, with the objective of developing a more robust controller [43,44] for closed-loop DBS applications.

## Figures and Tables

**Table 1:** Limitations of current techniques.

Frequency domain analysis	Frequency domain techniques identify artifact based on narrow frequency peaks in the frequency spectrum. The effectiveness of these methods depends on the chosen window size and threshold parameter C, which require careful selection and evaluation to achieve satisfactory results. DBS artifacts can be present across the entire frequency spectrum, including frequencies that are relevant for studying brain activity. Therefore, defining specific frequency bands for artifact removal may not be practical.
Template-based methods	Estimating a general template for DBS artifacts is challenging due to the wide variety of shapes they can have and their variability over time. Moreover, conventional template-based methods, which have primarily been used in single-pulse stimulation studies, may not yield optimal results when dealing with high-frequency DBS.
Low-pass and notch filters	Low-pass and notch filters may not be efficient if the stimulation peaks overlap other frequency bands.
Threshold-based methods	Underestimating the threshold can lead to a slight amplitude bias and potentially result in missing parts of the physiological data. Overestimating the threshold can leave residual artifacts.
Methods based on principal component analysis (PCA)	PCA can lead to the loss of information due to the reduction in dimensionality it induces.
Methods based on signal space separation (SSS)	SSS assumes that the natural brain activity is not correlated with any artifacts or unwanted signals. However, in reality, there can be situations where strong sources of activity leak into the intermediate part, leading to the false identification of sources as artifact.

**Table 2:** Power Differences between Original and Contaminated Signals Compared to Artifact-Free Signals in Different Frequency Bands: This comparison illustrates the reduction in artifacts in the time–frequency domain as a result of the artifact removal process.

	Average of Power Differences between	
	Original Signal and Contaminated Signal	Original Signal and Artifact-Free Signal
Delta band	0.1025	0.0469
Theta band	0.1026	0.0317
Alpha band	0.0485	0.0076
Beta band	0.0384	0.0552

**Table 3:** Time domain comparison of MSE values.

Technique	Mean-Squared Error (MSE) between the Original Signal and		Percentage Difference in MSE%
	Contaminated Signal (μV^2^)	Artifact-Free Signal (μV^2^)	
SVD	1230	58.57	−95.26
Gaussian model matching	1230	1540	+24.91
FIR wiener filtering	1230	671.55	−45.76
Adaptive filtering	1230	656.27	−46.99
Moving average	1230	1400	+16.01

**Table 4:** Comparison of Average Power for Various Filtering Techniques in Different Frequency Bands.

	Average Power					
	Baseline	SVD	Adaptive Filtering	FIR Wiener Filtering	Gaussian Model Matching	Moving Average
Delta	0.1930	0.1868	0.1427	0.0982	0.2805	0.0921
Theta	0.2246	0.2263	0.1797	0.1443	0.2875	0.1445
Alpha	0.1783	0.1880	0.1600	0.1621	0.1730	0.1697
Beta	0.2141	0.2198	0.2167	0.2760	0.1284	0.2866

## Data Availability

The data used in this study was published in [37] and is available within the same paper.
